# Identifying the Relationship Between Residential Type and Health Outcomes of the Community-Dwelling Thai Older Adults in the Baseline Analysis of a Cluster-Randomized Controlled Trial

**DOI:** 10.3390/geriatrics9060143

**Published:** 2024-11-05

**Authors:** Nadila Mulati, Myo Nyein Aung, Saiyud Moolphate, Thin Nyein Nyein Aung, Yuka Koyanagi, Siripen Supakankunti, Motoyuki Yuasa

**Affiliations:** 1 Department of Global Health Research, Graduate School of Medicine, Juntendo University, Tokyo 113-8421, Japan; m.nadila.vp@juntendo.ac.jp (N.M.); moyuasa@juntendo.ac.jp (M.Y.); 2 Faculty of International Liberal Arts, Juntendo University, Tokyo 113-8421, Japan; 3 Advanced Research Institute for Health Sciences, Juntendo University, Tokyo 113-8421, Japan; 4 Department of Public Health, Faculty of Science and Technology, Chiang Mai Rajabhat University, Chiang Mai 50300, Thailand; saiyud_m@cmru.ac.th; 5 Department of Family Medicine, Faculty of Medicine, Chiang Mai University, Chiang Mai 50200, Thailand; drthinnyeinaung@gmail.com; 6 Global Health and Chronic Conditions Research Group, Chiang Mai University, Chiang Mai 50200, Thailand; 7 Faculty of Health Sciences, Tokyo Ariake University of Medical and Health Sciences, Tokyo 135-0063, Japan; y-koyanagi@juntendo.ac.jp; 8 Centre of Excellence for Health Economics, Faculty of Economics, Chulalongkorn University, Bangkok 10330, Thailand; siripen.s@chula.ac.th

**Keywords:** healthy aging, active aging, built environment, gated communities, residential type, health promotion

## Abstract

Background/Objectives: As people age, their environment plays a critical role in shaping their health. With Thailand’s rapidly aging population, it is crucial to understand how different living environments affect the well-being of older adults. This study examines differences in biopsychosocial health indicators between older adults living in village communities and private housing estates in Chiang Mai, Thailand. Methods: A cross-sectional study was conducted using baseline data from the Community-Integrated Intermediary Care (CIIC) Service Model, a Cluster Randomized Controlled Trial in Thailand (TCTR20190412004). The study included 2788 older adults (aged 60+). Of these, 89.49% resided in village communities, and 10.51% in private housing estates. Validated instruments were used to assess health indicators. Descriptive statistics, multivariate analysis of variance, and multiple logistic regression analyses were performed. Results: Older adults in private housing estates had significantly lower odds of experiencing pain or discomfort (Adj OR: 0.64, 95% CI: 0.49–0.84) and were 1.36 times more likely to report positive perceived health. However, they had lower odds of perceiving themselves as physically and socially active (Adj OR: 0.74, 95% CI: 0.57–0.97) and were 0.30 times less likely to rate their quality of life higher (Adj OR: 0.30, 95% CI: 0.22–0.40) compared to their village community counterparts. Conclusions: The residential environment significantly influences older adults’ health and well-being. Tailored health promotion interventions should leverage the unique strengths of both village communities and private housing estates to enhance social connections, physical activity, and quality of life, promoting healthy, active aging across diverse settings.

## 1. Introduction

Countries worldwide are witnessing varying rates of growth in the proportion and count of older adults. Projections indicate that the global share of people aged 65 years or older will increase from 9.3 percent in 2020 to around 16.0 percent by 2050 [1]. Thailand, as the second most aging nation in the ASEAN region, is projected to rank among the top ten countries globally in terms of its older population, with approximately 27% of its population aged 60 years or older by the year 2030 [2]. This significant increase would result in Thailand ascending from its current 15th position to the 7th position in the global rankings [2].

The aging process is often accompanied by an increase in the amount of time individuals spend in their houses and neighborhoods due to factors such as retirement, a decrease in social activities, and various physical and social changes that come with aging [3]. Based on the general ecological model of aging, a person’s functioning is the result of their physical, psychological, and social resources, environmental factors, and the fit between ever-changing individuals and their ever-changing environments [4]. Physical health indicators of older adults, such as physical functions, disability rates, fall occurrences, health-related quality of life, and mortality rates, as well as mental and cognitive health aspects, including depression, anxiety, psychological well-being, and cognitive health, are all profoundly affected by the physical and social environment in which older individuals live [5,6,7,8,9,10]. This “context”, plays a critical role in shaping and determining the overall well-being and functioning of older adults [11].

Like most developing countries, the long-term care system in Thailand is community and family-based [12]. This means that the family and community members are the backbone for caregiving. The cultural norm that family members should take care of older adults in a household, and the lack of institutional care resulted in people remaining at their homes as they aged. This could result in the overburden of the informal caregivers, as well as the possible decrease in the quality of life of the care recipients [13]. Hence, in the context of healthy aging, it becomes imperative to delve into the concept of “aging in place”.

Urban development is accompanied by the emergence of enclosed urban spaces, for example, gated communities. It is defined as a walled or fenced housing development to which public access is restricted. It is characterized by legal agreements that tie the residents to a common code of conduct and (usually) collective responsibility for management [14]. Gated dwellings featured in their safety, privacy, and investment potential, gained popularity among people [14]. In Thailand, traditionally, people live in the “ban” or village communities, and has been considered as a fundamental social structure [15]. As the urban development started, gated communities were developed simultaneously. There are two types of gated communities: The low-rise or “*mubanchatsan*” (a cluster of detached houses) and the high-rise or “*aakanchut*” (the condominium, being a single high-rise residential building or cluster of them) [15]. Two types of residential settings have distinct health assets and liabilities that impact the well-being of older adults. On the one hand, a traditional village community offers a close social network, familiarity, and a sense of belonging among residents. These factors contribute to emotional support and social engagement, which are essential for mental well-being. On the other hand, emerging private housing estate communities offer a range of diverse amenities and enhanced physical infrastructure that promote mobility and physical activity. Additionally, these estates often feature improved security measures, which can enhance residents’ peace of mind and overall quality of life.

In the context of healthy aging, to our knowledge, there is no study examining the potential health difference among Thai older adults living in original village communities and gated communities yet. Given that Thailand is experiencing rapid population aging as a developing country, it is crucial to identify the factors that promote the health and well-being of older citizens, it is necessary to explore the housing type and well-being of older adults.

Therefore, the objective of our study is to examine the biopsychosocial indicators of community-dwelling older adults residing in the original community and private housing estate in Thailand. Specifically, we aim to address the following research questions: (1) How do health-related quality of life, functional ability, and mental state differ between older adults in traditional communities and those in private housing estates? (2) What variations exist in self-perceived health status, self-perceived quality of life, and self-perceived active aging among these two residential groups? We hope that the result of this research will contribute to evidence-based policy decisions and interventions regarding aging in the right place and improving the health and well-being of community-dwelling older adults in Thailand.

## 2. Materials and Methods

### 2.1. Study Design and Data Source

This is a cross-sectional study, using the baseline data from the “Community-Integrated Intermediary Care (CIIC) Service Model to Enhance Family-Based, Long-Term Care for Older Adults in Chiang Mai, Thailand: a cluster-randomized controlled Trial (TCTR20190412004)” [16]. It is a 2-arm, parallel-design, interventional study. The cluster is villages that have more than 300 older persons over 60 years of age at the time of randomization. A total of 12 clusters (25 villages) were included in this study [17]. The CIIC care model aimed to reduce the caregiver burden and promote the health of older adults by designing a service model, that consisted of providing support to caregivers to increase their care capacity, providing respite care service in the community, and providing physical exercise, mainly functional training to older adults [17].

### 2.2. Setting

The study was conducted in Chiang Mai, Northern Thailand. National statistics reveal that Chiang Mai Province is the third most populous province for old citizens, following Bangkok and Nakhon Ratchasima. Over 20% of the population in Chiang Mai Province are aged 60 years or older, which is higher than the national average of 18.24% [18]. In Thailand, life expectancy at birth increased by 4.04 years, from 71.3 years in 2000 to 75.3 years in 2021, while healthy life expectancy improved by 3.12 years, from 62.6 to 65.8 years over the same period [18].

### 2.3. Study Participants

According to the Thailand Older Persons Act, 2003, Article 3, the “Older Person” was defined as “a person who is sixty years of age” [19]. Male, and female individuals aged 60 years or over, residing in the study site with informed consent were included in this study. The total number of study participants was 2788 older adults. The sample size was calculated via STATA version 11SE, and the detail of sample size calculation was shown in the evaluation of the CIIC study [20]. Individuals who did not sign the informed consent, who cannot understand the explanation of informed content, and who have cognitive impairment or severe impairment of decision-making were excluded from this research.

### 2.4. Data Collection

The structured questionnaires were used to collect socio-demographic information, lifestyle habits, and biological, social, and psychological health status. The well-trained public health research assistants conducted door-to-door visits to the participants’ houses and collected the data according to the study protocol, and using an interview-administrated, structured questionnaire. The data collection period was six months in 2019 [17]. The collected data were entered using the double entry and validation method to ensure its quality.

### 2.5. Measures

The research instruments include: Thai versions of the EQ-5D-5L, Barthel’s Activities of Daily Living (ADL) index, and the Geriatric Depression Scale (GDS). All the instruments that were used in this study were already translated into the Thai language and validated in other aging and long-term care research [17]. In addition, pilot tests in the study area were conducted to ensure the reliability of the instruments in the study context. The overall reliability coefficients using Cronbach’s alpha of the EQ-5D-5L, ADL index and GDS were 0.89, 0.90 and 0.80, respectively.

#### 2.5.1. Outcome Variables

(1) Barthel Index of Activity of Daily Living (ADL)

The functional ability of the study participants was assessed using the Barthel Index of Activity of Daily Living (ADL). It is a standard measure used and validated in Thailand to assess the dependency status of older adults. It examines the ability to perform the ten daily basic activities, consisting of bathing, feeding, dressing, toilet use, transfer, indoor mobility, grooming, stairs, bowel control, and bladder control. The total possible scores range from 0 (maximal disability) to 20 (maximal independence) and are categorized into three categories: The total score equal to or more than 12 is categorized as mildly dependent to independent (coded as 0), 5~11 is categorized as moderately dependent (coded as 1), and the score of 0~4 categorized as severely dependent (coded as 2) [21].

(2) Euro-Qol five dimension five-level (EQ-5D-5L) index

To assess the health-related quality of life of the study participants, the Euro-Qol five dimension five-level (EQ-5D-5L) index (descriptive system questionnaire) and a visual analog scale (EQ VAS) was used [22]. The Thai version of the EQ-5D-5L index has already been validated and has been widely used [23,24]. It contained five-dimension, mobility, self-care, usual activities, pain/discomfort, and anxiety. Each dimension has five response levels: no problems (level 1), slight problems (level 2), moderate problems (level 3), severe problems (level 4), and unable to/extreme problems (level 5). The five dimensions were converted to a summary index (EQ-5D index) using a crosswalk calculator concerning the Thailand value set. According to the guideline, we dichotomized the EQ-5D levels into ‘no problems’ (level 1; coded as 0) and ‘any problems’ (levels 2, 3, 4, and 5; coded as 1). Moreover, the EQ Visual Analogue Scale (VAS) is a rating scale method to provide a global assessment of self-rated health status “today”, from 0 (worst imaginable health state) to 100 (best imaginable health state) we used the EQ-VAS to measure overall self-rated health status with a vertical VAS where the endpoints are labeled “Best imaginable health state” and “Worst imaginable health state” [22].

(3) Geriatric Depression Scale (GDS)

The state of depression was examined using the short form of the Geriatric Depression Scale (GDS) [25]. It is internationally, commonly, used to screen, diagnose, and evaluate depression in older adults [26]. The Thai version of the GDS scale was validated and regularly used in Thailand [27,28]. It consists of 15 items with “yes” or “no” responses, scored either as 0 points or 1 point. The total points are summed up to obtain the GDS total score, ranging from 0 to 15. A dichotomous scale of no depression (total GDS score 0–4; coded as 0), mild depression (total GDS score 5–10; coded as 1), having severe depression (total GDS score 11–15; coded as 2) was made to determine the depression among study participants [20].

(4) Self-perceived health status

Self-perceived health is a single-item question that has been used widely to examine the overall health of an individual [29,30]. Studies showed that self-perceived health status can be a predictorof mortality and morality [31,32,33]. In this study, we examined the perceived health status of older adults by using a 5-point scale, a single question: “In general, what do you think of your health condition?” The answer was “very bad”, “poor”, “not so poor”, “good”, and “very good”. There were very few participants who perceived their health as very bad, therefore, we categorized “very bad” and “poor” into “poor health status”, (coded as 0), “not so poor” as “neutral” (coded as 1), and “good”, “very good” to “good health status” (coded as 2).

(5) Self-perceived active aging

To examine how active the participants consider themselves with regards to physical activity and social participation, they were asked to rate from 1 to 10 how active their life is, 1 being the least active and 10 being the most active. This quantitative variable was not normally distributed, and the median of the self-rated active aging score was 8, hence the perceived active aging was categorized into being less active (<8) (coded as 0) and being more active (≥8) (coded as 1).

(6) Self-perceived quality of life

The study participants were asked to rate their quality of life from 1 to 10, 10 being the best quality of life and 1 being the lowest quality of life. This variable is non-normally distributed, and the median score was 7. Therefore, we categorized it into low quality of life (<7; coded as 0) and high quality of life (≥7; coded as 1).

#### 2.5.2. Measure of Independent Variables

The independent variable, the residential type was measured using a single question, the participants were either residing in the original village community or the housing estate community. The original community refers to the “ban” or village communities that were self-built housing [15]. The housing estate refers to a uniform, 40~200 cluster of gated houses developed by private real estate companies or property developers, with a range of amenities and facilities designed to provide a comfortable and convenient living environment for residents [15].

The socio-demographic information included age (completed years), was categorized into three categories, “60–69”, “70–79”, and “≥80 years old”, sex, marital status (“not married” including the single, separated, divorced, widowed, and “married” person), working status (“yes”, “no”), educational background (“no formal education”, including uneducated person, and not studied, but can read and write. “Primary school completed”, and “secondary and above education completed” consisted of people that gained secondary school education and/or vocational school/diploma, bachelor) and living arrangements (“living alone, “living with a spouse, and “living with children”, which includes son, daughter, son/daughters-in-law, and “living with others” includes siblings, grandchildren, relatives, and neighbors). The lifestyle factors including exercise (do not exercise, exercise but not regularly, and exercise regularly), smoking, and alcohol consumption (“never” and “quit” was categorized into “no”, and the answer “occasionally” and “always” was categorized into “yes”). Health conditions were diabetes mellitus (type 2), hypertension, and a history of falling in the past six months were explored.

### 2.6. Statistical Analysis

STATA version SE17 (Stata Corp 4905, Lakeway Drive, College Station, TX 77845 USA) was applied for data analysis. Descriptive analysis was conducted to analyze the sociodemographic information of the study participants. For scattered continuous variables, medians and interquartile range (IQR) was used. As for categorical variables, frequency and percentage were used. Bivariate analysis (Mann-Whitney test or Kruskal-Wallis test for continuous variables, Chi-square test, and Fisher’s exact test for categorical variables) was used to examine the statistical difference between biopsychosocial health indicators among housing estate and original community residents. Lastly, multivariable logistic regression was used to examine the potential correlation of residential type and different health indicators. *p* value less than 0.2 in bivariate analysis and conceptually related variables were included as potential confounders in the final regression model. Ordinal logistic regression was used for ADL, GDS, and self-perceived health status. Binary logistic regression analysis was used for EQ-5D-5L, self-perceived active aging, and self-perceived quality of life. Simple and multiple linear regression analysis was used for EQ VAS and EQ Index. All the health indicators were adjusted for sociodemographic characteristics (age, sex, educational background, marital status, working status, living arrangement), lifestyle behavior (smoking, alcohol consumption, and exercise behavior), and health states (diabetes, hypertension, history of falling), separately. Statistical significance was defined as *p* < 0.05 with a 95% confidence interval (CI). The fitness of each multiple logistic regression model was checked by applying the Hosmer–Lemeshow Goodness-of-fit test.

## 3. Results

### 3.1. The Characteristics of Study Participants

A total of 2788 Thai older adults were included in this study. The mean age of study participants was 69.69 years, most of them were female (60.11%), married (58.18%), and had completed primary school education (61.01%). Regarding the educational background of older residents, those in housing estates have a higher proportion (69.97%) of individuals who completed secondary school and above compared to those in the original community (27.86%). Most of the older adults (68.19%) were not working at the time the study was conducted. 10.29% of the participants were living by themselves, 33.93% of them were living with their spouse, 42.90% living with their children, and 12.88% of them living with others (Table 1).

Many of the participants in this study did not smoke (93.36%) or drink alcohol (79.23%). As for exercise behavior, 33.64% of the participants exercised regularly, 46.66% of them exercised, but not regularly, and 19.69% of the participants did not exercise at all. Among the individuals living in the original community, there was a slightly higher percentage (35.15%) of those who engage in regular exercise, compared to those residing in the housing estate (20.82%). There were 18.40% of study participants with diabetes and 48.71% of study participants with hypertension. A total of 280 people (10.04%) experienced a fall six months before the study period, and this percentage is slightly higher among older adults residing in the original community (10.26%) compared to those living in the housing estate (Table 1).

### 3.2. Association of Housing Type and Biopsychosocial Health Indicators

The differences in functional ability, health-related quality of life, psychological state, perceived health status, active aging, and quality of life among residents of the original community and housing estate were explored and the result is shown in Table 2. The proportion of older adults experiencing mobility problems and pain/discomfort was higher among those residing in the traditional community. However, these individuals perceived themselves as more active and reported a better quality of life compared to those living in private housing estates. In contrast, a higher percentage of participants from private housing estates had a more positive perception of their health status. These differences were statistically significant (*p* < 0.05).

There was no significant difference in functional ability between original community residents and housing estate residents. Most of the study participants (97.09%) were independent, which indicates that they were able to independently do daily activities such as mobility, getting dressed, going up-and-down from stairs, taking a bath, and using the toilet.

With regards to health-related quality of life, 77.91% of older adults had no problem walking about. Most of the older adults (90.96%) had no problem washing or dressing themselves, majority of the (88.02%) older adults had no problem doing their daily activities. Half of the study participants (50.47%) reported no pain or discomfort in their daily life and most of the older adults (89.17%) were not anxious or depressed. The mean EQ VAS score for study participants was 77.14 (SD 13.80), with no significant difference observed between original community and housing estate residents. However, when comparing health-related quality of life among different residential types, statistically significant differences were observed in the mobility (*p* = 0.002) and pain/discomfort (*p* < 0.001) dimensions, as well as in the EQ Index of the two groups (*p* < 0.001).

The study participants were in a good psychological health state, regardless of the residential type. The result showed that only 23 people (0.82%) had depressive disorder, majority of the people (92.22%) had no depression.

The perceived health status was statistically significantly different between the older adults residing in the original community and older adults residing in the housing estate (*p* = 0.003). 59.73% of the housing estate residents considered they were in a good, very good health state, 35.48% of them perceived in a neutral health state, and 4.78% perceived in a poor, very poor health state. This percentage was 49.50%, 43.41%, and 7.09%, for original community residents, respectively.

The self-perceived active aging status was different among these two groups. Nearly 60% of the housing estate residents thought they were less active than others, and this proportion was 40.36% for original community residents. The difference was statistically significant (*p* = 0.020).

More than half (56.27%) of the residents of the original community perceived higher quality of life and this proportion was 24.91% for residents of the housing estate, and this difference is statistically significant (*p* < 0.01) (Table 2).

### 3.3. Potential Association of Housing Type and Health Outcomes

The study investigated the correlation between housing type and the biopsychosocial indicators of community-dwelling Thai older adults. Older adults in private housing estates had significantly lower odds of experiencing pain or dis-comfort compared to those in village communities (Adj OR: 0.64, 95% CI: 0.49–0.84) and were 1.36 times more likely to report a positive perceived health status. However, they had lower odds of perceiving themselves as physically and socially active (Adj OR: 0.74, 95% CI: 0.57–0.97) and were 0.30 times less likely to rate their quality of life higher (Adj OR: 0.30, 95% CI: 0.22–0.40) compared to older adults in traditional village communities (Table 3).

The health-related quality of life showed no significant association with housing type, except for the pain/discomfort dimension. Older adults living in the housing estate had significantly lower odds of experiencing pain/discomfort compared to those living in the original community (Adj OR: 0.64, 95% CI: 0.49, 0.84) (Table 3). Moreover, a statistically significant relationship between housing type and the EQ Index was found before adjusting for the covariates. Living in a housing estate was associated with a higher EQ Index. However, when considering the additional factors (covariates) in the multiple linear regression analysis, this relationship lost its statistical significance. There was no indication of the correlation between housing type and EQ VAS (Table 4).

Housing type was significantly associated with perceived health status (Adj OR: 1.36, 95% CI: 1.04, 1.77). Specifically, older adults living in the housing estate associated with 1.36 times higher odds of reporting a more positive perceived health status (good, very good) compared to older adults in the original community (Table 3).

The perception of active aging and quality of life were correlated with the type of housing. Older adults residing in housing estates were found to have 0.74 times lower odds of perceiving themselves as active in terms of physical activity and social engagement compared to those living in the original community (Adj OR: 0.74, 95% CI: 0.57, 0.97). Additionally, they had 0.30 times lower odds of rating their quality of life higher (Adj OR: 0.30, 95% CI: 0.22, 0.40) (Table 3).

## 4. Discussion

Aging does not occur in a vacuum. It is shaped by the environment, circumstances, and choices that have been made. A supportive and empowering residential and neighborhood environment that encourages independence, autonomy, movement, engagement, and participation promotes healthy aging [34,35,36,37,38].

This study result showed the complex interplay between residential type and the health indicators of community-dwelling Thai older adults. Living in a private housing estate (gated community) demonstrates a positive association with reduced pain/discomfort and improved perceived health status. Conversely, older adults living in original village community were more inclined to perceive themselves as active both physically and socially, along with reporting a higher quality of life.

Older adults are more prone to experience both acute and chronic pain compared to the younger generation [39]. Our study showed that older adults living in the original community are more likely to experience pain/discomfort compared to the housing estate older residents (Table 3). Older age is associated with negative health-related quality of life in mobility, self-care, usual activities, and pain/discomfort dimensions [40]. In this study, the original community group had a higher percentage of oldest-old individuals (aged 80 or more) compared to the housing estate group (Table 1), this might explain the high probability of experiencing pain/discomfort in original community residents. Moreover, a supportive environment that has adaptive features, a mobility-friendly neighborhood, etc. can help with managing pain [41]. Although no statistically significant difference in mobility, self-care, and usual activity was found, more older adults in the original community reported problems in these dimensions compared to those in the housing estate (Table 2). Housing estate communities are equipped with better quality amenities, and higher quality housing, that might contribute to better pain management, less likelihood of experiencing pain/discomfort, and less restriction in mobility and usual activity among its older residents.

Regarding self-rated quality of life, older adults in the original community tend to report higher levels compared to those in housing estates (Table 3). Before accounting for sociodemographic factors and other variables (as shown in Table 4), the type of residence was significantly linked to health-related quality of life, indicating that those in housing estates reported a higher quality of life in terms of health. The quality of life differs from the health-related quality of life that focuses on measuring the connection between health and quality of life [42]. It is defined by the World Health Organization “as an individual’s perception of their position in life in the context of the culture and value systems in which they live and in relation to their goals, expectations, standards, and concerns [43]. Health is only one dimension of quality of life [44]. Residents of the original community are surrounded by close social connections, with familiar neighborhoods, with many families living in the same area for generations. Older adults in these communities frequently retain some form of community role, providing them with a sense of purpose. This strong network and sense of contribution might be a facilitator toward a better-perceived quality of life for original village community residents [45].

The older people living in the housing estate are more likely to perceive a positive health status compared to those living in the original community (Table 3). The health of an individual is defined as “a state of complete physical, mental and social well-being and not merely the absence of disease or infirmity” [46]. Self-perceived health status is a proxy for not only the physical health, but also psychological, social, and emotional health of an individual [47]. Satisfaction with community services significantly contributed to the positive perceived health state [48]. The gated communities are equipped with good-quality amenities and more convenient access to goods and services. Furthermore, within our study, there was a higher representation of younger individuals (aged 60–69) in the housing estate group in comparison to the original community group, and they exhibited a higher level of educational attainment than the residents of the original community, as shown in Table 2, residents of gated communities are economically wealthier as well. These socio-economic factors may exert an influence on the overall health status of older adults [49,50].

Active aging is defined as “the process of optimizing opportunities for health, participation, and security to enhance the quality of life as people age” [51]. Being “active”, does not solely refer to being physically active, but also refers to active involvement in social, economic, cultural, spiritual, and civic aspects [51]. The study result showed that compared to those living in housing estates, older adults in the original community had higher odds of perceiving themselves as active. Health, participation, and security are the three basic pillars of active aging. Participation refers to engaging in formal or informal work, and volunteer activities based on the needs, interests, and capacity [52]. The older adults residing in the original community had a higher percentage of working people (Table 1). Additionally, research within the same setting indicated that those in the original community were more inclined to participate in community-based group exercise programs than their counterparts in the housing estate [53]. Familiarity with the local environment contributes to activity participation [5]. Hence, residents of the original community showed a stronger perception of being physically and socially active.

The health indicators mentioned above may be interconnected. The improved health-related quality of life among housing estate residents could be attributed to their more positive perceptions of their health state. Conversely, the original community residents’ perception of being physically and socially more active may have contributed to their better self-rated quality of life.

Our results suggest that policies should address the distinct needs of different residential environments, with targeted approaches to promote the well-being of older adults. In private housing estates, where social isolation may be more common, efforts should focus on creating opportunities for social engagement through community centers, organized activities, and programs that encourage intergenerational interactions, and foster stronger connections among residents. In contrast, for village communities, where social ties are already strong but physical health outcomes may lag, interventions should emphasize accessible healthcare services, physical activity programs, and infrastructure improvements that encourage mobility. This could include the development of age-friendly public spaces, such as walking paths and exercise facilities, as well as health promotion campaigns tailored to the local population. By considering these distinct needs, policies can effectively promote both social engagement and physical health, ensuring that older adults, in all types of residential environments, are physically, psychologically, and socially healthy. This study has several strengths and limitations. The study site, Chiang Mai, is the most populous province in Northern Thailand. Research conducted in this context, focusing on residential types and the well-being of older adults, can offer valuable insights that have the potential to inform evidence-based policies and interventions aimed at promoting healthy and active aging, considering factors related to the built environment. Our study makes a significant contribution towards this goal, in part due to the relatively large sample size and the meticulous data collection process conducted through door-to-door interviews. However, the association between the residential type and the health and well-being of the older population should be studied in the future as the longitudinal studies. This would provide greater clarity on how changes in residential environments influence health outcomes over time, offering a more precise understanding of causal relationships. Moreover, it could track shifts in physical, social, and mental well-being, enabling policymakers to develop targeted interventions that evolve with the aging population’s needs.

## 5. Conclusions

In conclusion, this study provides valuable insights into the relationship between residential type and various health-related indicators among older adults. Our study highlights the nuanced ways in which housing type may influence older adults’ perceptions of activity, quality of life, and health status. These findings have implications for future research and the development of targeted interventions to enhance the well-being of older adults in different residential settings. There are contrasting opinions regarding the living environment for older adults. Some advocate for staying in the same house and neighborhood, emphasizing the preservation of social connections, increased independence, and autonomy. Conversely, others suggest that residing in upgraded neighborhoods can positively impact older individuals’ health due to improved living conditions, safety, and convenience. Considering the heterogeneity of the older adults, and the findings from this study, we conclude that there is no one-size-fits-all solution when it comes to the built environment and the well-being of older adults. Both the original community and housing estate have their advantages and disadvantages, depending on the values, sense of belonging and expectation of the older adults. Therefore, the intervention and programs aimed at enhancing the health and well-being of older adults should consider the distinct characteristics of older residents across various residential neighborhoods. Furthermore, the active involvement of older residents in the design of age-friendly environments and neighborhood planning is vital to developing tailored solutions that meet the diverse needs and preferences of this population.

## Figures and Tables

**Table 1:** Sociodemographic status and lifestyle behavior of study participants (N = 2788).

	Original Community	Housing Estate	Total
	N (%)	N (%)	N (%)
Study Participants	2495 (89.49%)	293 (10.51%)	2788 (100%)
Age Mean (SD)	69.69 (8.17)	68.24 (7.35)	69.54 (8.10)
Age Group			
60–69	1475 (59.12%)	194 (66.12%)	1669 (59.86%)
70–79	669 (26.81%)	72 (24.57%)	741 (26.58%)
≥80	351 (14.07%)	27 (9.22%)	378 (13.56%)
Sex			
Male	1005 (40.28%)	107 (36.52%)	1112 (39.89%)
Female	1490 (59.72%)	186 (63.48%)	1676 (60.11%)
Marital Status			
Not married (single, divorced, widow)	1038 (41.60%)	128 (43.69%)	1166 (41.82%)
Married	1457 (58.40%)	165 (56.31%)	1622 (58.18%)
Educational Background			
No formal education	178 (7.13%)	9 (3.07%)	187 (6.71%)
Primary school completed	1622 (65.01%)	79 (26.96%)	1701 (61.01%)
Secondary school and above completed	695 (27.86%)	205 (69.97%)	900 (32.28%)
Current Work Status			
Not working	1683 (67.45%)	218 (74.40%)	1901 (68.19%)
Working	812 (32.55%)	75 (25.60%)	887 (31.81%)
Living Arrangement			
Alone	252 (10.10%)	35 (11.95%)	287 (10.29%)
Spouse	861 (34.51%)	85 (29.01%)	946 (33.93%)
Children	1064 (42.65%)	132 (45.05%)	1196 (42.90%)
Others	318 (12.75%)	41 (13.99%)	359 (12.88%)
Smoking			
No (never, quit)	2321 (93.03%)	282 (96.25%)	2603 (93.36%)
Yes (occasionally, always)	174 (6.97%)	11 (3.75%)	185 (6.64%)
Alcohol Consumption			
No (never, quit)	1973 (79.08%)	236 (80.55%)	2209 (79.23%)
Yes (occasionally, always)	522 (20.92%)	57 (19.45%)	579 (20.77%)
Exercise Behavior			
Do not exercise	480 (19.24%)	69 (23.55%)	549 (19.69%)
Exercise, but not regularly	1138 (45.61%)	163 (55.63%)	1301 (46.66%)
Exercise regularly	877 (35.15%)	61 (20.82%)	938 (33.64%)
Diabetes			
No	2020 (80.96%)	255 (87.03%)	2275 (81.60%)
Yes	475 (19.04%)	38 (12.97%)	513 (18.40%)
Hypertension			
No	1267 (50.78%)	163 (55.63%)	1430 (51.29%)
Yes	1228 (49.22%)	130 (44.37%)	1358 (48.71%)
History of Falling in the Past Six Months			
No	2239 (89.74%)	269 (91.81%)	2508 (89.96%)
Yes	256 (10.26%)	24 (8.19%)	280 (10.04%)

Note: SD = Standard deviation.

**Table 2:** Bivariate analysis of outcome variables with different residential types.

Outcome Variables	Original Community N (%)	Housing Estate N (%)	Total N (%)	*p*-Value
The Activity of Daily Living				
Mean (SD)	19.24 (2.56)	19.19 (2.57)	19.23 (2.56)	
Mildly dependent on independent	2423 (97.11%)	284 (96.93%)	2707 (97.09%)	0.916
Moderately dependent	43 (1.72%)	6 (2.05%)	49 (1.76%)	
Severely dependent	29 (1.16%)	3 (1.02%)	32 (1.15%)	
Health-related quality of life				
Mobility				0.002
No Problem	1923 (77.07%)	249 (84.98%)	2172 (77.91%)	
Any Problem	572 (22.93%)	44 (15.02%)	616 (22.09%)	
Self-Care				0.238
No Problem	2264 (90.74%)	272 (92.83%)	2536 (90.96%)	
Any Problem	231 (9.26%)	21 (7.17%)	252 (9.04%)	
Usual Activities				0.177
No Problem	2189 (87.74%)	265 (90.44%)	2454 (88.02%)	
Any Problem	306 (12.26%)	28 (9.56%)	334 (11.98%)	
Pain/Discomfort				<0.001
No Problem	1219 (48.86%)	188 (64.16%)	1407 (50.47%)	
Any Problem	1276 (51.14%)	105 (35.84%)	1381 (49.53%)	
Anxiety/Depression				0.959
No Problem	2225 (89.18%)	261 (89.08%)	2486 (89.17%)	
Any Problem	270 (10.82%)	32 (10.92%)	302 (10.83%)	
EQ-5D Index mean (SD)	0.80 (0.23)	0.86(0.23)	0.81 (0.23)	<0.001
EQ-VAS Scores mean (SD)	77.06 (13.76)	77.75 (14.18)	77.14 (13.80)	0.279
Geriatric Depression Scale				
Mean (SD)	1.42 (2.19)	1.25 (2.19)	1.41 (2.19)	
Normal	2300 (92.18%)	271 (92.49%)	2571 (92.22%)	0.425
Mild depression	176 (7.05%)	18 (6.14%)	194 (6.96%)	
Depressive disorder	19 (0.76%)	4 (1.37%)	23 (0.82%)	
Self-perceived health status				0.003
Good, very good	1235 (49.50%)	175 (59.73%)	1410 (50.57%)	
Neutral	1083 (43.41%)	104 (35.49%)	1187 (42.58%)	
Poor, very poor	177 (7.09%)	14 (4.78%)	191 (6.85%)	
Self-perceived active aging				
Mean (SD)	7.72 (1.57)	7.42 (1.91)	7.68 (1.61)	
Less active (<8)	1007 (40.36%)	1488 (59.64%)	1146 (41.10%)	0.020
More active (≥8)	1488 (59.64%)	154 (52.56%)	1642 (58.90%)	
Self-perceived quality of life (QOL)				
Mean (SD)	6.86 (1.58)	5.73 (1.09)	6.75 (1.57)	
QOL < 7	1091 (43.73%)	220 (75.09%)	1311 (47.02%)	<0.001
QOL ≥ 7	1404 (56.27%)	73 (24.91%)	1477 (52.98%)	

Note: SD: standard deviation.

**Table 3:** Multivariable logistic regression analysis of health outcomes and residential type (N = 2788).

Dependent Variables	Functional Ability (ADL)	EQ-5D-5L					Geriatric Depression Scale	Self-Perceived Health Status	Self-Perceived Active Aging	Self-Rated Quality of Life
		Mobility	Self-Care	Usual Activities	Pain/Discomfort	Anxiety/Depression				
	Adj OR (95% CI)	Adj OR (95% CI)	Adj OR (95% CI)	Adj OR (95% CI)	Adj OR (95% CI)	Adj OR (95% CI)	Adj OR (95% CI)	Adj OR (95% CI)	Adj OR (95% CI)	Adj OR (95% CI)
Original community	Referent	Referent	Referent	Referent	Referent	Referent	Referent	Referent	Referent	Referent
Housing estate	1.52 (0.70, 3.30)	0.74 (0.51, 1.06)	0.93 (0.56, 1.54)	0.93 (0.59, 1.44)	0.64 ** (0.49, 0.84)	1.20 (0.79, 1.84)	1.22 (0.75, 2.00)	1.36 * (1.04, 1.77)	0.74 ** (0.57, 0.97)	0.30 ** (0.22, 0.40)

Note: Adj OR = Adjusted Odds Ratio. * *p* < 0.05. ** *p* < 0.01.

**Table 4:** Simple/multiple linear regression analysis of health-related quality of life of older adults in different housing types.

	EQ VAS		EQ Index	
	Unadj. Coeff (95% CI)	Adj. Coeff (95% CI)	Unadj. Coeff (95% CI)	Adj. Coeff (95% CI)
Original community	Referent	Referent	Referent	Referent
Housing estate	0.68 (−0.99, 2.36)	0.28 (−1.41, 1.97)	0.06 ** (0.03, 0.08)	0.03 (0.00, 0.06)

Note: Unadj. Coeff = Unadjusted Coefficient; Adj. Coeff = Adjusted Coefficient; ** *p* < 0.01.

## Data Availability

The datasets generated during and/or analyzed during the current study are not publicly available due to the privacy of the study participants, but are available from the corresponding author on reasonable request.
